# Supplementary material for: Comparative genomics provides new insights into the diversity, physiology, and sexuality of the only industrially exploited tremellomycete: Phaffia rhodozyma
Source: BMC Genomics. 2016 Nov 9;17:901. doi: 10.1186/s12864-016-3244-7 (PMC5103461; doi:10.1186/s12864-016-3244-7)
Supplement: Additional file 6: — List of orphan genes with links to PFAM (related to Additional file 1: Table S1). (ZIP 1428 kb) [file 12864_2016_3244_MOESM6_ESM.zip › PFAM_UNIQUE/G01889_P.html]

#                                                                            --- full sequence --- -------------- this domain -------------   hmm coord   ali coord   env coord
  
# target name        accession   tlen query name           accession   qlen   E-value  score  bias   #  of  c-Evalue  i-Evalue  score  bias  from    to  from    to  from    to  acc description of target
  
#------------------- ---------- ----- -------------------- ---------- ----- --------- ------ ----- --- --- --------- --------- ------ ----- ----- ----- ----- ----- ----- ----- ---- ---------------------
  
G01889\_P             -            582 Atg31                PF09795.4    160      0.15   10.4   0.0   1   3      0.41   2.5e+03   -3.3   0.0   130   153    79   102    68   107 0.74 - Autophagy-related protein 31  
G01889\_P             -            582 Atg31                PF09795.4    160      0.15   10.4   0.0   2   3         1     6e+03   -9.2   6.9    48    62   209   221   205   228 0.42 - Autophagy-related protein 31  
G01889\_P             -            582 Atg31                PF09795.4    160      0.15   10.4   0.0   3   3   2.6e-05      0.15   10.4   0.0    42    95   372   423   368   434 0.80 - Autophagy-related protein 31
